# Enhancing the therapeutical potential of metalloantibiotics using nano-based delivery systems

**DOI:** 10.3762/bjnano.16.98

**Published:** 2025-08-15

**Authors:** Alejandro Llamedo, Marina Cano, Raquel G Soengas, Francisco J García-Alonso

**Affiliations:** 1 Nalón Innova, Avenida de Galicia 31, 33005, Oviedo, Spain; 2 Departamento de Química Orgánica e Inorgánica, Instituto Universitario de Química Organometálica “Enrique Moles”, Universidad de Oviedo, Julián Clavería 8, 33006 Oviedo, Spain

**Keywords:** antimicrobial resistance, biocompatibility, metalloantibiotics, nanocarriers, targeted delivery

## Abstract

The rapid spread of antibiotic resistance has intensified the need for novel therapeutic strategies against multidrug-resistant bacterial infections. Metalloantibiotics present a promising alternative in combating resistant pathogens. However, the clinical application of metalloantibiotics is limited by their potential toxicity, instability, and lack of target specificity. Encapsulating metalloantibiotics in drug delivery systems, such as liposomes, nanoparticles, and polymeric carriers, could mitigate these challenges, enhancing their therapeutic index and enabling their precise, localized release. Recent reviews have outlined the key design parameters and clinical translation challenges associated with nanocarrier-based antimicrobial therapies, underscoring their relevance in overcoming bacterial resistance mechanisms [Xie, Y.; Liu, H.; Teng, Z.; Ma, J.; Liu, G. *Nanoscale* **2025,** *17,* 5605–5628. https://doi.org/10.1039%2FD4NR04774E ]. This review explores the potential of encapsulated metalloantibiotics as a new frontier in antimicrobial therapy. We address the mechanisms by which drug delivery systems can stabilize and direct metalloantibiotics to their biological targets, discuss current advancements in encapsulation methods, and examine the efficacy of encapsulated metalloantibiotics. Finally, we consider the challenges and future directions for the integration of metalloantibiotic-loaded carriers in the fight against antibiotic-resistant infections.

## Introduction

Antimicrobial resistance (AMR), the condition that bacteria no longer respond to drugs used to treat infections, has become one of the biggest public health challenges of the 21st century. According to a study by Antimicrobial Resistance Collaborators, AMR was associated with 4.95 million deaths globally in 2019, highlighting its immense impact on public health. Without immediate and sustained efforts to improve treatment of infections, it is estimated that, by 2050, antimicrobial resistance could cause 1.91 million deaths each year, and that a further 8.22 million people will die from illnesses associated with resistance [1]. Different reviews further highlight the evolving complexity of antimicrobial resistance and underscore the need for multidisciplinary strategies to overcome it [2–3].

This global crisis arises from a combination of the abuse and misuse of antibiotics, the lack of new antibiotic drugs in clinical development, and the remarkable ability of microorganisms to adapt and evolve. Antimicrobial resistance can be partly explained by natural selection, which allows bacteria to develop mutations that reduce or completely eliminate the antibiotic efficacy [4]. If bacteria are exposed to non-lethal quantities of an antibiotic, these bacteria will eventually develop resistance to the antibiotic [5]. Moreover, bacteria have the ability to transfer resistance genes directly between themselves through plasmids, promoting the rapid spread of antimicrobial resistance [6]. In this regard, the abuse of antibiotics in livestock and agriculture has been identified as one of the main factors that have led to the emergence of AMR [7].

The emergence and spread of AMR has intensified the interest in discovering new active antimicrobial compounds, as the current pipeline is incapable of addressing the urgency of this issue [8]. This concern has also been echoed in recent analyses evaluating AMR trends and antibiotic development strategies [9]. Most of the antibiotics currently in clinical development are derivatives of already existing ones, which makes them susceptible to existing resistance mechanisms [10]. In 2024, the World Health Organization (WHO) identified only 32 antibiotics under development to address the priority pathogens [11], of which only twelve fulfil all criteria to be considered as fully innovative, that is, no cross-resistance, new chemical class, new target, and new mechanism of action. Furthermore, just four of them are active against at least one pathogen of the top risk category [12].

The urgent need for novel antibiotics has led researchers to consider repurposing as a strategy to discover new antibiotic drugs [13]. In this context, the gold(I) antirheumatic drug Auranofin (Ridaura^TM^) [14] was evaluated for its antibacterial activity, exerting potent antimicrobial effect against multiresistant strains [15]. The promising antibacterial profile of auranofin sparked the interest in the design of antibiotic drugs that include metal ions in their structure, the so-called metalloantibiotics [16–17]. Metal-based antibiotics offer significant advantages over purely organic drug candidates in the fight against AMR [18]. In metal complexes, the wide range of oxidation states, coordinating ligands, and geometries yield access to a highly underexplored chemical space for antibiotic drug development [19]. Moreover, metalloantibiotics may provide unique and multiple modes of action including redox activation and catalytic generation of toxic species (reactive oxygen species, ROS), exchange or release of ligands, abolition of key enzyme activities, disruption of membrane function, and damage of the bacterial DNA [20].

Despite the obvious advantages of metal-based over purely organic drugs, there are several potential limitations that must be overcome for gold metalloantibiotics to reach clinical application [21]. One of the main characteristics of metallodrugs is their low aqueous solubility; this, combined with their typically short in vivo half-lives, results in inadequate bioavailability and low accumulation at the therapeutic site [22]. Another limiting factor is the reputation of metal-based drugs as toxic agents, related to studies that describe systemic toxicity of several metallodrugs, mainly related to liver damage and cardiotoxic effects [23]. In this sense, several recent studies show that encapsulation in nanotechnological systems is a general solution to overcome these problems [24]. Thus, encapsulation systems create a protective environment for metallodrugs, ensuring that they are delivered to the therapeutic site intact and limiting their interaction with healthy cells. In this way, nanoencapsulation systems drastically improve the efficacy and safety of metallodrug treatments [25].

While several recent reviews have addressed broad nanotechnology-based strategies against antimicrobial resistance, this work focuses specifically on the therapeutic potential of encapsulated metalloantibiotics [26]. It provides an overview of the properties and mechanisms of nanotechnology-based drug delivery systems, followed by their integration with metal-based complexes. Finally, it discusses the challenges and future perspectives of this emerging field, emphasizing its potential to revolutionize the fight against bacterial infections and antimicrobial resistance.

## Review

### Nanocarriers for targeted antibiotic delivery

The role of drug delivery systems is particularly vital in combating bacterial infections where antibiotic resistance poses a significant challenge [27–28]. Conventional antibiotic treatments often result in sublethal drug concentrations at infection sites, contributing to the emergence of multidrug-resistant (MDR) bacteria [29]. In contrast, new nanosystems aim to control the rate, time, and location of antibiotic release, modulating the pharmacokinetics effect at the desired site of action [30–31]. Nanotechnology offers innovative solutions to the challenges of traditional drug delivery methods, enhancing the ability of therapeutic agents to bypass systemic and local barriers, thereby improving precision and efficiency in reaching target sites [32–34]. These barriers include systemic, microenvironmental, and cellular obstacles that hinder the delivery of sufficient drug concentrations to the infection site [33].

Systemic barriers refer to challenges associated with the pharmacokinetics of antibiotics, such as rapid clearance, nonspecific distribution, and interactions with healthy tissues [35]. These factors are responsible for having a diluted concentration of antibiotics at the site of infection, necessitating higher dosages that increase the risk of side effects [36–37]. To address the abovementioned barriers, delivery systems often incorporate targeting strategies to improve accumulation at infection sites. Targeted delivery mechanism are broadly categorized into passive and active targeting [38].

Passive targeting is controlled by size, charge, and composition of the nanoparticle, which influences the localization, cell penetration, and release of the drug as physicochemical features of pathogenic tissues facilitate the drug accumulation. Optimization of these parameters helps to maximize efficacy and mitigate toxicity of the payload [39]. Infection sites often present unique microenvironmental features, a factor that nanoparticles exploit to achieve precise therapeutic delivery. For example, polymeric nanoparticles constructed with pH-sensitive polymers can be engineered to degrade in acidic environments, such as those found at infection sites. Additionally, certain bacteria at these sites express enzymes like lipase and hyaluronidase, which can be leveraged to design enzyme-sensitive antibiotic delivery systems [40–41]. Other bacteria, such as *Staphylococcus aureus* express alpha-toxin, which perforates the membrane of liposomes and can be utilized to trigger the release of antibiotics from the drug delivery system at the target site [42].

**Figure 1:**
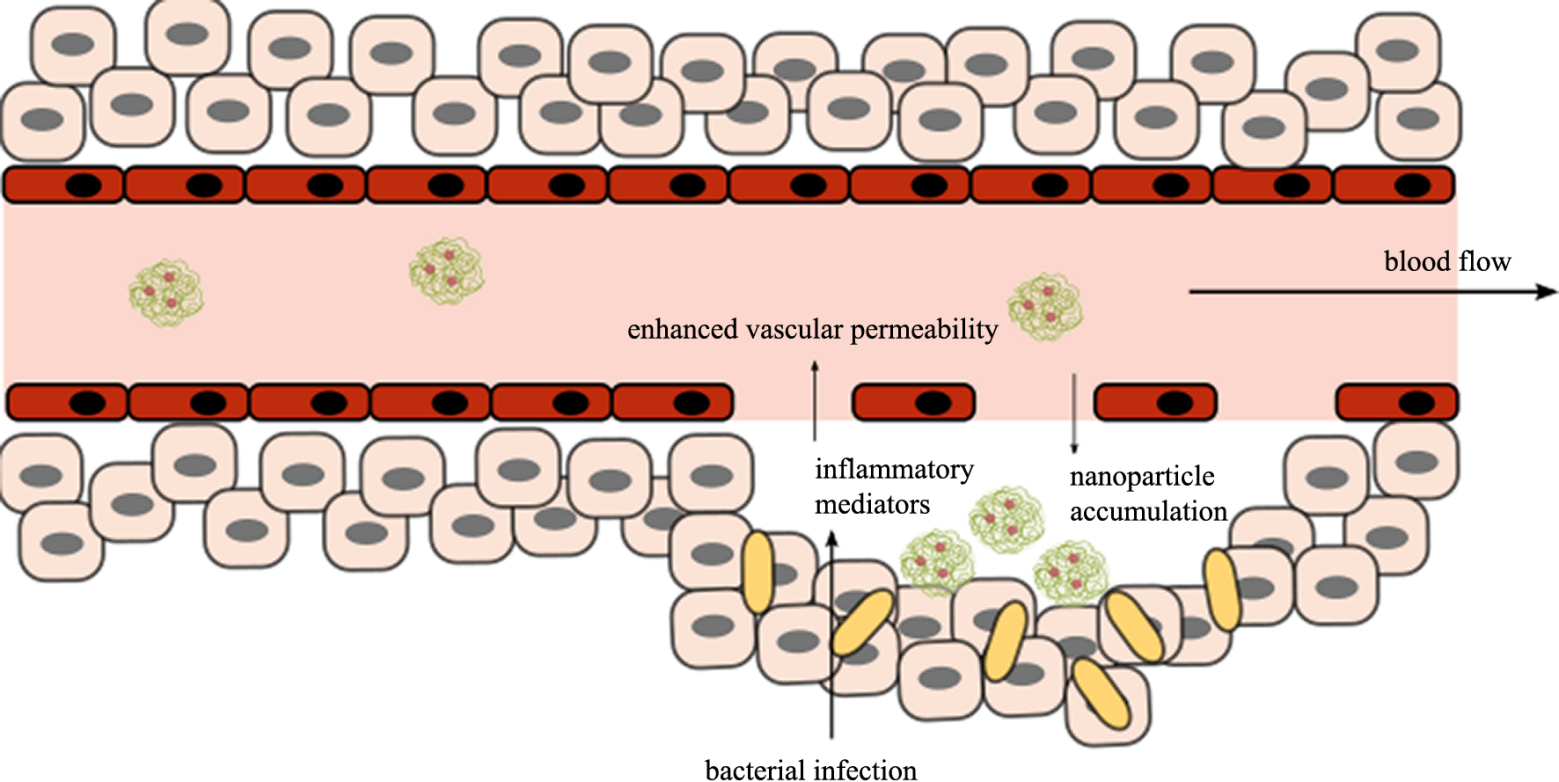
The role of the EPR effect in infections can be leveraged for targeted delivery anti-infective therapies.

Also, enhanced permeability and retention (EPR) is observed at the site of infections (Figure 1). After bacteria enter the body, lipopolysaccharides from Gram-negative bacterial cell walls and lipoteichoic acid from Gram-positive bacterial cell walls stimulate the immune system and provoke the liberation of inflammatory mediators, which subsequently enhance vascular permeability. Due to the intrinsic characteristics of nanoparticles, they can accumulate at infection sites due to this effect, resulting in better therapeutic outcomes and reduced toxic problems [43–44].

In contrast to passive targeting, active targeting takes advantage of the conjugation of drug carriers with ligands that bind to specific receptors overexpressed on the surface of the target site [45]. There are notable differences between the surfaces of eukaryotic cells and pathogenic bacteria, which provides obvious advantages in active-targeting strategies. In Gram-positive bacteria, antibiotics can bind to multiple sites in the cell wall peptidoglycans (PGNs), resulting in inhibition of PGN synthesis, perturbation of the cell membrane integrity and cell death [46–47]. In Gram-negative bacteria, lipopolysaccharides interact electrostatically with positively charged antibiotics [48] and bacterial lectins could serve as effective binding sites for glycosylated polymers [49]. Targeted nanoparticles need to be designed with an optimal density of targeting moieties to effectively interact with specific cell surface receptors. Achieving this requires a clear understanding of the ratio between receptors and ligands, as well as the number of interactions necessary to overcome the energy barrier for cellular uptake. Properly balancing these factors ensures efficient binding and internalization of the nanoparticles by the target cells [50–51]. For example, nanoparticles can be engineered to interact with upregulated mannose receptors on macrophage surfaces during inflammation. Mannosylated polymeric ligands have been developed for targeted delivery of antibacterial drugs to macrophages, leveraging the high-affinity interaction with mannose receptors on these immune cells [52]. Additionally, studies have demonstrated that mannose receptor-targeted rifampicin delivery through solid lipid nanoparticles (SLNs) can be effectively applied to the treatment of infections, highlighting the role of polymer-based systems in enhancing drug delivery to macrophages [53]. Both passive and active targeting approaches take advantage of the specific features of infection sites to enhance the precision of antimicrobial delivery.

**Figure 2:**
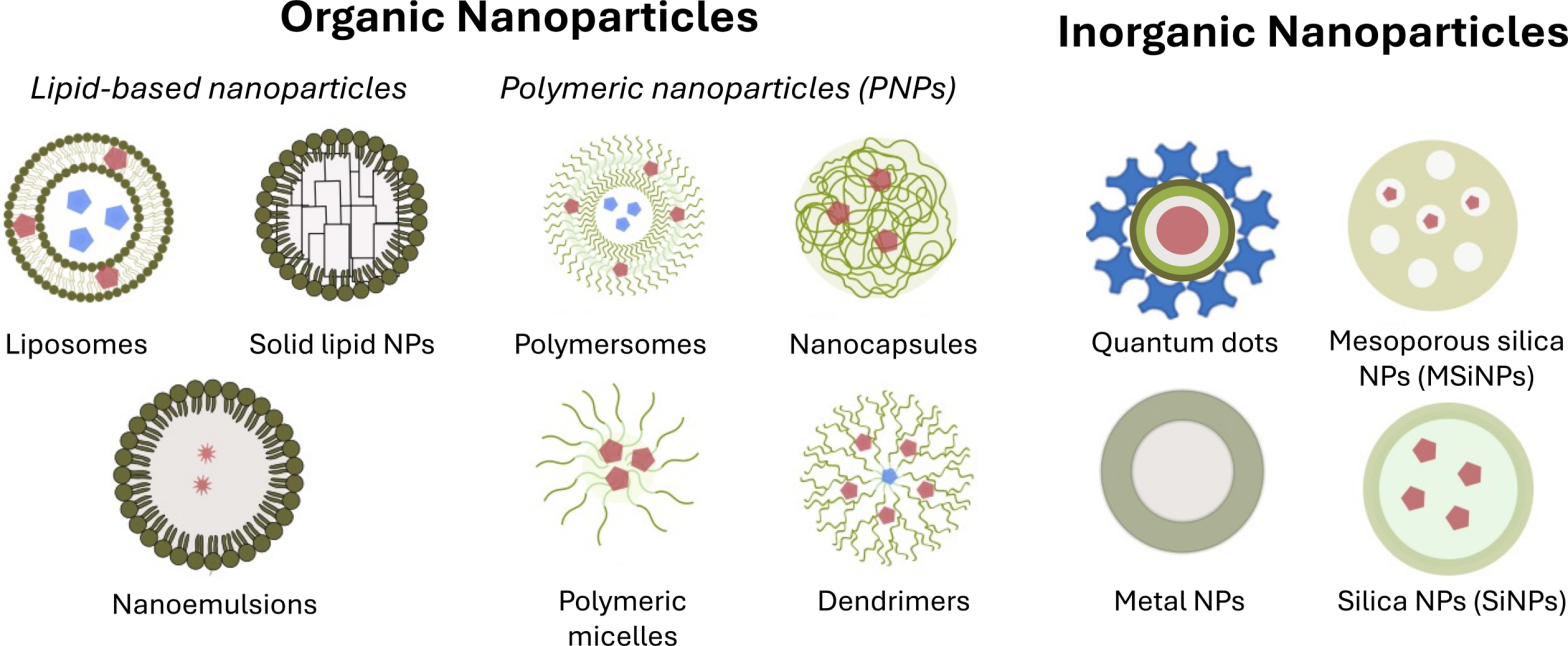
Most relevant drug delivery systems used in anti-infective therapies.

In summary, the incorporation of antibiotics into various nanocarriers represents a promising strategy to address bacterial resistance. These systems can improve therapeutic efficacy by enabling controlled release and selective accumulation at the infection site, as well as facilitating the delivery of antibiotics to intracellular bacterial reservoirs [54–55]. Altogether, these advantages contribute to enhanced therapeutic efficacy, improved solubility of poorly water-soluble compounds, and the possibility of co-delivering multiple active agents within a single system [56]. Since different encapsulation materials provide distinct features in drug encapsulation and delivery, the selection of suitable encapsulation materials is crucial and depends on the nature of the drug, the targeted therapeutic effect, and the desired release rate. The most common encapsulation nanosystems are represented in Figure 2.

### Organic nanoparticles

**Lipid-based nanoparticles.***Liposomes:* Liposomes are versatile lipid-based nanoparticles that have gained prominence in drug delivery systems due to their ability to carry both hydrophilic and hydrophobic drugs. These self-assembled vesicles consist of phospholipid bilayers that encapsulate an aqueous core, allowing for the entrapment of various therapeutic agents [57]. Liposomes can be designed to carry multiple types of drugs within the same system, significantly expanding their applications [58]. The structure of liposomes can vary, with unilamellar and multilamellar vesicles being the two main forms, offering flexibility in the types of drugs they can deliver. In addition, size, surface charge, and lipid composition of these nanoparticles can be tailored during synthesis, making them adaptable for different drug delivery needs [59–61].

Surface modifications, such as the incorporation of polyethylene glycol (PEG) or specific targeting ligands, are commonly used to enhance liposome circulation time and promote targeted drug delivery. These modifications prevent rapid uptake by the reticuloendothelial system, prolonging the presence of the drug in the bloodstream and increasing drug levels in the central nervous system [62]. Additionally, functionalizing liposomes with ligands or antibodies can facilitate specific interactions with cell surface receptors, allowing for precise targeting to the site of infection or tumor. This ability to focus drug delivery at the desired location enhances therapeutic efficacy while minimizing side effects [63].

The versatility of liposomes extends to their role in improving the efficacy of various antibiotics. For instance, liposomal formulations of antibiotics such as polymyxin B, cefepime, and vancomycin have demonstrated superior antibacterial effects compared to their free drug counterparts [64–66]. Liposomes enhance drug stability, increase drug concentration at the target site, and facilitate drug uptake by phagocytic cells, which is particularly beneficial for treating infections with biofilm-forming bacteria [67–69].

*Solid lipid nanoparticles:* SLNs are sub-micrometer colloidal carriers, typically ranging from 50 to 1000 nm, composed of lipids that remain solid at room and body temperatures. These nanoparticles consist of a solid lipid core matrix stabilized by emulsifiers in an aqueous dispersion [70–72]. SLNs were developed as an alternative nanocarrier system to emulsions, liposomes, and polymeric nanoparticles, combining the advantages of traditional liposomal and polymeric systems, such as biocompatibility and drug protection, while avoiding many of their limitations. In comparison to conventional colloidal carriers, SLNs demonstrate reduced toxicity, greater surface area, enhanced biocompatibility, extended drug release, improved cellular uptake, and increased drug solubility and bioavailability [71]. They are formulated from lipids that are generally recognized as safe, making them highly biocompatible and low in toxicity [72]. Moreover, the solid lipid matrix slows down drug diffusion and lipid degradation, enabling a controlled, long-lasting release of the encapsulated drug [70]. SLNs are capable of encapsulating both hydrophilic and lipophilic drugs, and their surface can be functionalized to enhance circulation or tissue-specific delivery [72–73]. Nowadays, SLNs are used as a carrier for several chemotherapeutic drugs including antibiotics. Common methods for drug encapsulation in SLNs include high-pressure homogenization and microemulsion techniques. In these processes, drugs are incorporated in the melted lipid before nanoparticle formation, leading to high entrapment efficiency within the solidified lipid matrix, whereas hydrophilic drugs may be partitioned at the interface or within imperfections of the solid core [74].

Despite their advantages, SLNs face certain challenges, such as limited drug loading capacity due to the crystalline structure of the solid lipid matrix, risks of drug leakage during storage, and a relatively high water content in the formulation [73]. To address these issues, researchers have developed nanostructured lipid carriers, which incorporate liquid lipids into a solid lipid matrix, resulting in improved drug loading capacity and stability [75].

In summary, SLNs have garnered significant attention as a promising drug delivery system, combining high biocompatibility, prolonged release properties, and the capacity to encapsulate a diverse range of therapeutic agents. Ongoing research efforts are focused on overcoming existing limitations to fully realize the clinical potential of SLNs [76].

**Polymer nanoparticles.** Polymeric nanoparticles (NPs) are highly versatile drug delivery systems that can be synthesized from natural or synthetic materials, providing a broad range of possible structures and characteristics. These NPs are typically fabricated through techniques such as emulsification, nanoprecipitation, or microfluidics, and can encapsulate a wide variety of therapeutic agents, from hydrophilic to hydrophobic compounds. Due to their biocompatibility and easy formulation, polymeric NPs can be engineered to offer precise control over drug release, stability, and targeting, making them ideal candidates in the treatment of infections [77].

The most common forms of polymeric NPs include nanocapsules, which have a polymeric membrane enclosing the drug payload, and nanospheres, consisting of a solid polymer matrix. Among these, polymersomes and dendrimers are notable types of polymeric NPs [78]. Polymersomes, made from amphiphilic block copolymers, exhibit good stability and cargo-retention efficiency, making them ideal for cytosolic drug delivery [79]. Polymeric micelles, with a hydrophilic core and hydrophobic outer shell, protect aqueous drug cargo and improve circulation time, often being used for the delivery of cancer therapeutics [80]. Dendrimers, with their hyperbranched structures, can be precisely controlled for size, shape, and surface chemistry, allowing for highly targeted delivery of anti-biofilms drugs or nucleic acids [81–82].

Polymeric NPs offer several advantages, including biodegradability, biocompatibility, and stability during storage [83–84]. Their surfaces can be modified for targeted delivery, improving the bioavailability of therapeutic agents and allowing for their use in a variety of medical applications. Despite these advantages, challenges such as particle aggregation and potential toxicity continue to persist. Nevertheless, polymeric NPs are undergoing extensive testing in clinical trials and are considered promising candidates for future drug delivery systems [85–86].

### Inorganic nanoparticles

**Silica nanoparticles.** Silica nanoparticles (SiNPs) are widely used in drug delivery due to their high surface area and the presence of polar silanol groups, which enhance stability and the ability to absorb water. These properties make them excellent carriers for bioactive molecules, providing efficient encapsulation and controlled release. SiNPs can be engineered to release their payloads in response to specific stimuli, making them ideal for targeted drug delivery applications [87].

Mesoporous silica nanoparticles (MSiNPs) are a subclass of SiNPs known for their ordered pore structure, which allows for the encapsulation of large molecules and proteins. Due to their high surface area for drug loading and also to the fact that the pore size can be precisely controlled during synthesis, MSiNPs are widely applied for delivering drugs to targeted sites, particularly in cancer and infectious diseases [88–89]. In addition, MSiNPs can be further enhanced by surface modifications, such as the attachment of polyethyleneimine, which improves cellular uptake and facilitates drug release within the target site [90]. The incorporation of stimuli-responsive agents in the pores also enables MSiNPs to release therapeutic agents upon exposure to specific environmental cues, such as pH or temperature. These interesting features for targeted release, coupled with the ability to efficiently deliver drugs to cells like macrophages, make MSiNPs an attractive option for overcoming drug delivery challenges, such as those encountered with bacterial infections [91].

**Quantum dots.** Quantum dots (QDs) are semiconductor nanocrystals generally ranging between 1 to 10 nm in size. They have recently garnered attention as drug delivery systems due to their ability to easily penetrate cell membranes and their large specific surface area, which facilitates extensive drug conjugation and precise targeting [92–93]. These nanoparticles exhibit size-dependent fluorescence due to quantum confinement effects, enabling precise tuning of emission wavelengths [94]. These properties make QDs ideal candidates for both imaging and drug delivery. Unlike other nanoparticles, QDs do not encapsulate drugs internally; instead, they function as drug delivery systems by attaching therapeutic molecules to their surface [95].

The surface of QDs can be engineered to carry multiple functionalities simultaneously, such as targeting ligands, PEG chains for stealth, and drug molecules [94]. When conjugated with antibiotics, QDs can enhance drug uptake by bacteria, improve biofilm penetration, and increase antibacterial efficacy, offering a promising strategy against resistant bacterial strains [96]. Recent developments in the design of heavy metal-free QDs along with biocompatible surface coatings have also significantly reduced concerns regarding toxicity, thus improving their prospects for clinical translation [97]. While QDs holds great potential for antibiotic drug delivery, challenges such as achieving precise targeting and ensuring long-term photostability remain critical limitations [98].

### Encapsulation of metalloantibiotics in nano-based delivery systems

Encapsulation of metal complexes within advanced nanoparticles has shown immense potential in antimicrobial therapy [99]. Thus, there are several recent studies reporting that these drug delivery systems can overcome the limitations in the therapeutic use of free metalloantibiotic drugs, including low selectivity, poor biodistribution and pharmacokinetics, poor water solubility, dose-limiting toxicity, and fast degradation in vivo. We will discuss next the major discoveries in the field of the application of nano-based systems for the targeted delivery of metal complex-based antibiotic compounds, focusing on the most relevant elements.

#### Silver complexes

Silver complexes have been extensively studied for their antimicrobial properties, which are mainly attributed to the high solubility of silver(I) ions. Among them, silver-*N*-heterocyclic carbene (NHC) complexes are particularly relevant as potential antimicrobial agents since they usually exhibit superior antimicrobial activity compared to silver(I) alone [100]. The mechanism of action of most silver complexes is based on a slow release of the silver(I) ions, which react with the thiol groups of proteins or with key functional groups of enzymes; the coordinated ligands merely serve as carrier for silver(I) ions. Additionally, silver ions can also generate ROS, which target primarily lipids, DNA, RNA and proteins, leading to serious consequences [18]. Despite a complete understanding of the mechanisms of antibacterial action is yet to be achieved, it has been suggested that the antibacterial action is strongly related to bioavailability and stability. In fact, the poor water solubility, the degradation (precipitation) by chloride in the bloodstream, the interaction with sulfur-containing proteins, and the rapid clearance by macrophages are the main factors limiting the systemic use of silver metalloantibiotics [101]. To overcome these limitations, several nano-based delivery systems were investigated.

**Figure 3:**
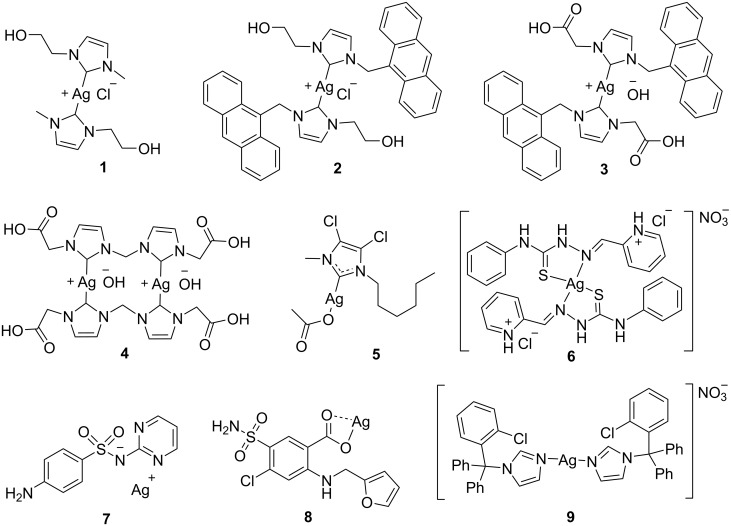
Nano-encapsulated antibacterial silver complexes.

For example, the silver–*N*-heterocyclic carbene (Ag-NHC) complexes **1**–**4** (Figure 3) were successfully encapsulated in amphiphilic block copolymer micelles [102]. The nano-encapsulated complexes maintained their antibacterial properties, improving solubility and stability. In addition, reduced toxicity and side effects were observed for the nano-encapsulated Ag-NHC complexes compared to the free metallodrug. Additionally, the encapsulation system prevented the aggregation of silver ions, which is crucial for maintaining the antibacterial activity of the silver complexes.

The Ag-NHC complex **5** (Figure 3) was also encapsulated in ʟ-tyrosine nanoparticles, which offer enhanced therapeutic potential through controlled and targeted release [103]. This encapsulation demonstrated significant efficacy in vitro against *P. aeruginosa*, a bacterial pathogen relevant to cystic fibrosis. Further in vivo evaluation revealed that the lungs of the mice treated with the encapsulated silver complex appeared quite normal and the concentration of bacteria in them was significantly reduced by the aerosol application. Collectively, in vitro and in vivo studies demonstrated that these nanoparticles achieve sustained release of the active drug species over several days, leading to a notable survival advantage in mouse infection models with only two doses. This sustained release profile not only enhances antimicrobial effectiveness but also holds strong clinical potential by reducing dosing frequency, thereby improving patient compliance.

Poly(ε-caprolactone) was yet another polymeric structure used for the encapsulation of Ag-NHC complexes. Thus, encapsulation of Ag-NHC complex **6** (Figure 3) resulted in nanosystems that not only exhibit suitable particle sizes and ζ-potentials for drug delivery but also have improved antibacterial efficacy compared to the free Ag metallodrug. In this regard, the minimum inhibitory concentration (MIC) and the minimum bactericidal concentration (MBC) of the encapsulated Ag complex decreased five-fold compared to those of the free metallodrug. Additionally, the encapsulated compound inhibited *Helicobacter pylori* biofilm formation while reducing free silver complex toxicity to mammalian cells and mitigating its mutagenic effects [104].

Dellera et al. developed wound dressings containing silver sulfadiazine **7** and platelet lysate encapsulated in SLNs to treat persistent skin lesions. The Ag-encapsulating SLNs were prepared using ultrasound and hot homogenization techniques and incorporated into chitosan glutamate or hydroxypropylmethyl cellulose-based dressings. The resulting formulation displayed improved antimicrobial activity and enhanced wound healing properties [105]. Additionally, in vitro assays revealed that encapsulating silver sulfadiazine in SLNs protected fibroblasts and keratinocytes from metal-induced toxicity. Finally, the coatings also exhibited excellent biocompatibility, elasticity, hydration, and bioadhesion properties [106].

Encapsulation into SLNs was also used to enhance antibacterial activity and achieve sustained release of the silver–furosemide (FSE) complex **8** (Figure 3) [107]. Although the free complex showed potent antibacterial effects, its efficacy is limited by its poor solubility in water and most organic solvents. Ag-FSE-loaded SLNs demonstrated high encapsulation efficiency (≈93%) and drug loading (≈9.3%), with a spherical shape and smooth surface. In vitro release studies confirmed that encapsulation into SLNs resulted in sustained release of complex **8** over a 96-hour period. Additionally, the antibacterial activity was significantly improved, with a twofold increase against *P. aeruginosa* and a fourfold increase against *S. aureus*. These findings suggest that Ag-FSE-loaded SLNs hold promise for the formulation of topical antibacterials agent for treating bacterial infections.

Finally, the clotrimazole silver complex **9** (Figure 3) was encapsulated in SLNs aiming at improving the activity against methicillin-susceptible and methicillin-resistant *S. aureus* [108]. Due to the ability of these nanoparticles to control drug release, the nanoencapsulation of clotrimazole silver complex enhances and prolongs the antibacterial activity.

#### Gold complexes

Gold compounds have garnered significant attention in recent years for their diverse clinical applications. Widely recognized for their anti-rheumatic and anticancer activities, recent studies also highlight their increasing potential as antibacterial agents [17]. Regarding the mechanisms of action, gold complexes inhibit thioredoxin reductase, explaining the selectivity of several gold complexes to Gram-positive strains [109]. Furthermore, some gold complexes can interact directly with cell membranes causing their destabilization [110].

**Figure 4:**
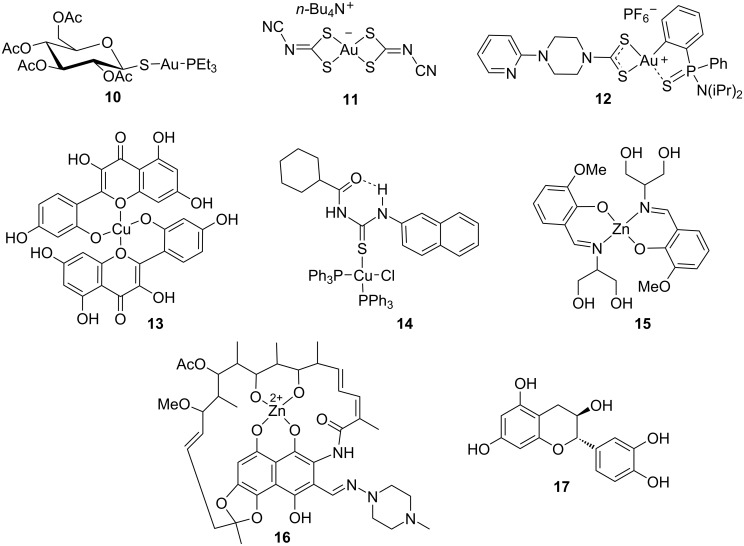
Nano-encapsulated antibacterial gold, copper, and zinc complexes.

As stated before, the FDA-approved antirheumatic gold(I)–phosphine complex auranofin **10** (Figure 4) is the reference metalloantibiotic, displaying a potent bactericidal activity against drug-resistant Gram-positive bacteria, such as *S. aureus*, *E. faecium*, *E. faecalis*, and *M. tuberculosis*.

In order to achieve a sustained release of auranofin to the infection site and improve the treatment efficacy, Díez-Martínez et al. investigated the encapsulation of this Au(I) complex in poly(lactic-*co*-glycolic acid) (PLGA) nanoparticles and the antibacterial efficacy of the resulting formulation against the Gram-positive pathogens *S. pneumoniae* and *S. pyogenes* [111]. Auranofin-PLGA NPs exhibited a strong bactericidal effect, effectively sterilizing multiresistant pneumococcal strains at a Au(I) concentration of just 0.25 μM. This potent bactericidal effect was also observed in *S. pneumoniae* and *S. pyogenes* biofilms, where the same concentration of auranofin NPs reduced bacterial populations by approximately four orders of magnitude more than free auranofin. The antibacterial effects observed in vitro were further confirmed in vivo using a zebrafish embryo model, showing improved survival rates against pneumococcal infections on treatment with auranofin-loaded NPs compared to free auranofin.

In another example, the gold(III) bisdithiolate complex **11** (Figure 4) was encapsulated in block copolymer micelles (BCMs) to improve solubility, bioavailability, and therapeutic effectiveness [112]. The resulting Au-loaded BCMs exhibited a high loading efficiency and uniform size and displayed improved activity against *S. aureus* and *C. glabrata* compared to the free gold metallodrug.

Also, our group described a novel lipoformulation encapsulating the gold(III) metalloantibiotic **12** (Figure 4) in liposomes [113]. The Au-loaded liposomes displayed high loading efficiency, high stability, and particle size and zeta potential values suitable for drug delivery. Even though the antibacterial activity against resistant strains of *S. aureus* was not improved, a steep decrease in the cytotoxicity of the liposomal formulation compared to free gold(III) metalloantibiotic was observed, resulting in an optimal therapeutic index. The formulation also minimized gold-induced cardiotoxicity and cytochrome inhibition, addressing some of the key limitations of gold-based drugs.

#### Copper complexes

Copper complexes have garnered attention due to their diverse applications, owing to their antioxidant, antiviral, antibacterial and anticancer properties [114]. Many studies attribute the antibacterial activity of copper to its capacity to release ions that can disrupt bacterial cell membranes. Following membrane degradation, copper-released ions penetrate into the bacterial cell causing oxidative stress by production of ROS and subsequent degradation of the DNA [114–115].

Recent research has focused on the antibacterial properties of copper complexes derived from quinolones [116]. These complexes are recognized for their interaction with DNA gyrase enzymes like topoisomerases I and II, inhibiting their normal function and converting them into DNA-damaging agents. Ternary complexes of fluoroquinolones, copper(II), and phenanthroline have been developed to address bacterial resistance mechanisms that involve reduced membrane permeability. These complexes may bypass conventional porin-mediated pathways, enabling drug entry through alternative mechanisms, primarily controlled by electrostatic interactions with the membrane surface [117].

In 2022, Ghosh et al. described the encapsulation of the antimicrobial morin-Cu(II) complex **13** (Figure 4) in human serum albumin (HSA) or PLGA NPs [118]. The resulting nanoencapsulated systems displayed spherical morphology and high encapsulation efficiency, with average particles sizes of 214 ± 6 nm for Mor-Cu-HSA-NPs and 185 ± 7.5 nm for Mor-Cu-PLGA-NPs. High negative zeta potential values suggested that both encapsulation systems are sufficiently stable for drug delivery. In addition, both systems exhibited a biphasic release pattern, with an initial burst release followed by sustained and controlled release of the morin-Cu(II) complex. Finally, the antibacterial activities of both nanoformulations were higher compared to those of morin-Cu(II) complex, especially in the case of Mor-Cu-PLGA-NPs.

More recently, the antibacterial Cu(I) acylthiourea complex **14** (Figure 4) was incorporated into polycaprolactone/lignin (PCL/Lig) electrospun nanofiber composites, resulting in materials with promising antimicrobial properties [119]. One of the composites exhibited a stable and continuous release of the Cu(I) complex during the first 12 h, followed by a slower but controlled release for up to 20 days. Antibacterial efficacy tests against *E. coli* and *B. subtilis* revealed that the Cu-loaded composites were active against Gram-positive bacteria but ineffective against Gram-negative strains. This difference is likely due to the hydrophobic nature of the nanofiber membranes, which may hinder interaction with Gram-negative bacteria.

#### Zinc complexes

Zinc complexes have demonstrated remarkable activity against both Gram-positive and Gram-negative bacteria. While zinc complexes show promise in their free form, their full potential is often limited by solubility, stability, and targeted delivery challenges. Encapsulation addresses these issues by providing controlled release, reducing toxicity to non-target cells, and protecting the active compound from degradation in physiological environments. For example, emulsions incorporating lipophilic Zn(II) complexes have demonstrated sustained release over 24 h in diffusion assays, paving the way for advanced topical antibacterial treatments [120].

Different nanosystems have been explored to enhance the effectiveness of zinc complexes, including their incorporation into silica NPs. In this context, the Zn(II)–Schiff base complex **15** (Figure 4) was encapsulated in sol–gel-derived silica nanoparticles, and the resulting formulation was evaluated regarding its antimicrobial activity against Gram-positive bacteria (e.g., *S. aureus* and *B. subtilis*) and Gram-negative strains (e.g., *E. coli* and *P. aeruginosa*) [121]. Unfortunately, encapsulation led to reduced immediate efficacy compared to the free Zn complexes.

In another example, a nano-based delivery system was developed comprising the zinc–rifampicin complex (Zn-RIF) **16** (Figure 4) encapsulated within transferrin-functionalized silver quantum dots (Zn-RIF-Tf-QDs) [122]. This formulation markedly enhanced antimycobacterial activity, with at least a tenfold increase in efficacy against *M. smegmatis* and *M. bovis* relative to the free Zn complex. Immunofluorescence analyses confirmed selective uptake of Zn-RIF-Tf-QDs by macrophages and dendritic cells, with minimal internalization by lung epithelial cells and no evidence of cytotoxicity or genotoxicity. Another important aspect is that conjugates were localized within LAMP-1-positive late endosomal compartments, supporting their capacity for sustained intracellular drug release.

β-Chitosan nanoparticles (β-CS-NPs) can also be used to encapsulate antimicrobial zinc complexes, as reported by Zhang and colleagues. Thus, the complex formed upon treatment of the flavonoid catechin (CAT, **17**) (Figure 4) and a Zn(II) salt was encapsulated in β-CS-NPs, and the antibacterial performance against *E. coli* and *L. innocua* was subsequently investigated [123]. Nano-encapsulated systems exhibited low polydispersity and high positive surface charges suitable for drug delivery. Notably, CAT–Zn-loaded β-CS-NPs displayed significantly enhanced antibacterial activity, with MIC and MBC values as low as 0.031 and 0.063 mg/mL, respectively. The system maintained good stability under acidic conditions (pH 2.0–4.5), further supporting its potential as a functional antibacterial platform.

#### Ruthenium complexes

Ruthenium-based metal complexes have been extensively studied, and some of them have shown notable antimicrobial activity [124–125]. This activity can be attributed to their strong binding affinity for nucleic acids and proteins, ligand exchange kinetics comparable to those of platinum complexes, their two predominant oxidation states (II and III), and their ability to mimic iron when interacting with biological molecules [124]. However, their therapeutic application is hindered, as in most of the metallodrugs, by poor water solubility, low stability in aqueous solutions under physiological conditions, and unfavorable metabolic or biodistribution profiles.

**Figure 5:**
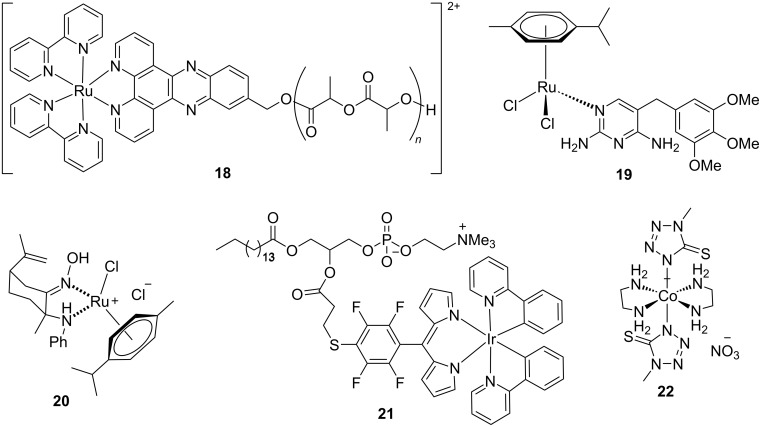
Nano-encapsulated antibacterial ruthenium, iridium, and cobalt complexes.

**Figure 6:**
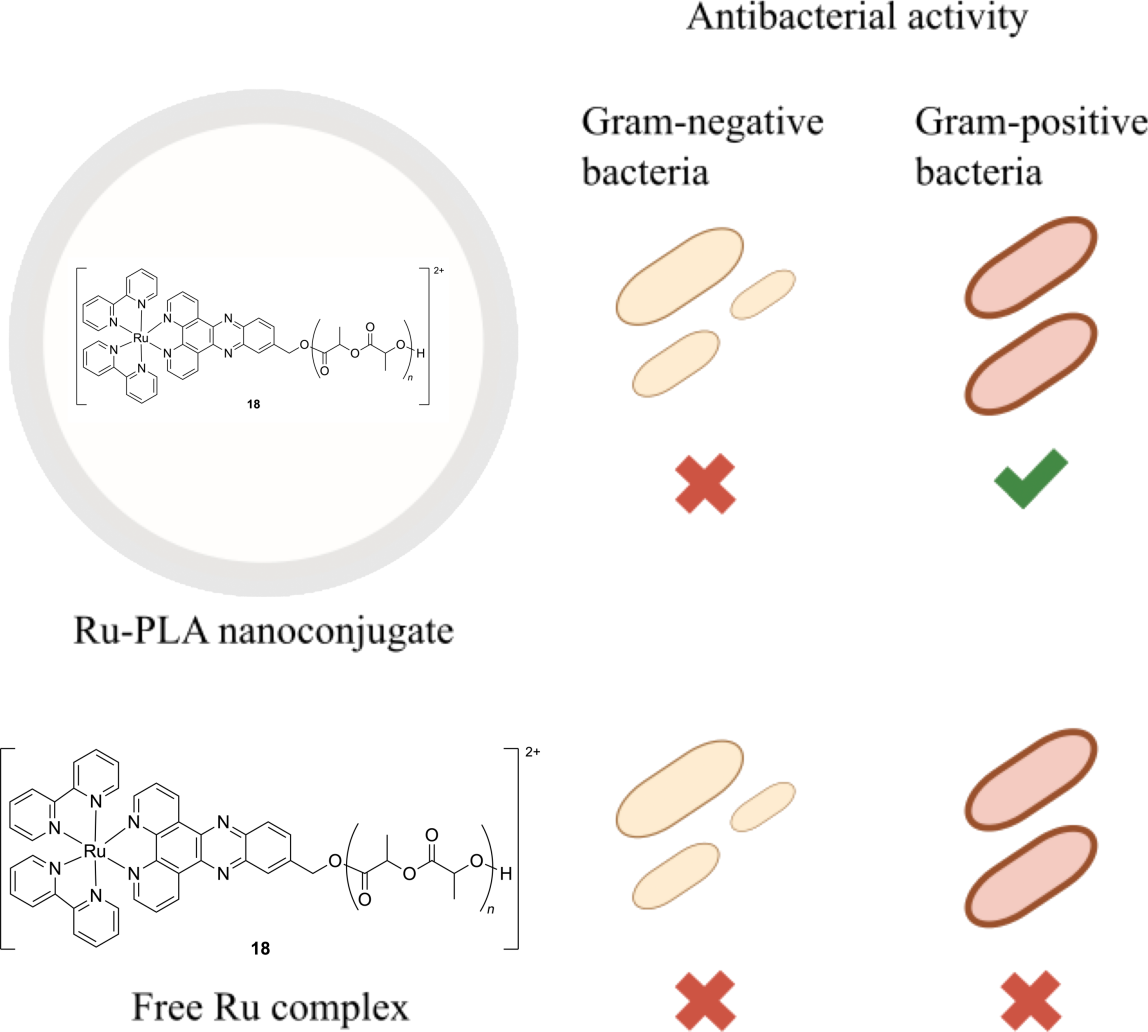
Antibacterial activity of Ru(II)-PLA nanoconjugates compared to free Ru(II) complex.

To address these limitations and enhance the delivery of Ru metalloantibiotics to the infection site, several nano-based strategies were developed [126]. For example, Gasser and co-workers disclosed the encapsulation of the Ru(II) polypyridyl complexes **18** (Figure 5) in nanoconjugates of poly(lactic acid) (PLA) with varying molecular weights [127]. Nanoprecipitation produced narrowly dispersed nanoparticles with high ruthenium loadings (up to 53%), as confirmed by dynamic light scattering. The antibacterial activity of these nanoparticles was evaluated against Gram-positive and Gram-negative bacterial strains (*S. aureus, S. epidermidis, E. coli*, and *P. aeruginosa*) and compared to the free complex **18**. While neither free drug **18** nor the encapsulated metallodrug showed activity against Gram-negative bacteria, Ru-PLA nanoconjugates displayed moderate bactericidal activity against Gram-positive strains (Figure 6).

Liposomes are also suitable nano-based delivery systems for antibacterial Ru complexes. A relevant example is the lipoformulation of the ruthenium complex **19** composed of the antibacterial drug trimethoprim and a well-known anticancer Ru fragment (Figure 5) [128]. While encapsulation did not affect the antibacterial activity of the Ru complex against *P. aeruginosa* and *S. aureus*, the systemic toxicity of the complex was significantly reduced, improving the therapeutic potential of the Ru metalloantibiotic.

Yet another promising nano-based strategy to improve the selectivity index of Ru complexes is microencapsulation. For example, Khelissa et al. reported the microencapsulation of the water-soluble ruthenium(II) complex **20** comprising bioactive aminooxime ligands derived from (*R*)-limonene (Figure 5) [129]. The resulting formulation was tested for antibacterial and antibiofilm activity against four foodborne pathogens, namely, *E. coli*, *S. aureus*, *L. monocytogenes*, and *E. faecalis*, displaying enhanced lower MICs compared to the free drug, especially against *E. coli*, *S. aureus*, and *L. monocytogenes*. In addition, improved biofilm disruption and reduced cytotoxicity were also observed.

#### Bismuth complexes

Bismuth and its derivatives have been widely employed in biomedical applications [130]. Among their most prominent applications is the treatment of *Helicobacter pylori* infections, a major contributor to peptic ulcer disease [131]. More recently, bismuth citrate has been shown to suppress replication of SARS-CoV-2 [132], the virus responsible for the COVID-19 pandemic, highlighting the expanding therapeutic potential of bismuth-containing compounds across diverse infectious diseases.

A relevant example of the use of nano-based delivery systems to improve the therapeutic potential of bismuth metalloantibiotics is the development of a liposomal formulation co-encapsulating bismuth-ethanedithiol and tobramycin (LipoBiEDT-TOB), designed to enhance antibiotic delivery and overcome bacterial resistance in *P. aeruginosa* and *B. cenocepacia* [133], opportunistic Gram-negative pathogens that represent a major therapeutic challenge in cystic fibrosis patients due to their intrinsic resistance mechanisms and biofilm-forming capabilities [134–135]. In vitro antimicrobial testing of the liposomal formulation against clinical isolates revealed a remarkable reduction in MIC and MBC when compared to free tobramycin. Notably, the formulation eradicated a highly resistant *P. aeruginosa* strain at concentrations 1000-fold lower than the free antibiotic. The enhanced activity was attributed to the synergistic effect of the bismuth complex and tobramycin, as well as the ability of liposomes to facilitate intracellular delivery. Furthermore, LipoBiEDT-TOB effectively inhibited bacterial adhesion of *B. cenocepacia* to lung cells, supporting its potential to interfere with early stages of infection and biofilm formation.

#### Iridium complexes

Antimicrobial photodynamic therapy (aPDT) is gaining recognition as a promising alternative to traditional antibiotics for managing chronic skin infections [136]. However, its broader application is contingent upon the development of highly selective and efficient photosensitizer delivery systems. Ir(III) complexes are excellent photosensitizers due to their highly sensitive excited-state properties in response to the surrounding environment, high photostability, and unique intracellular localization [137–138]. However, the significant intrinsic cytotoxicity of Ir(III) complexes remains a limiting factor for biomedical applications; in this regard, the development of nanocarriers that can enhance the biocompatibility of these complexes is currently of much interest [139–140].

**Figure 7:**
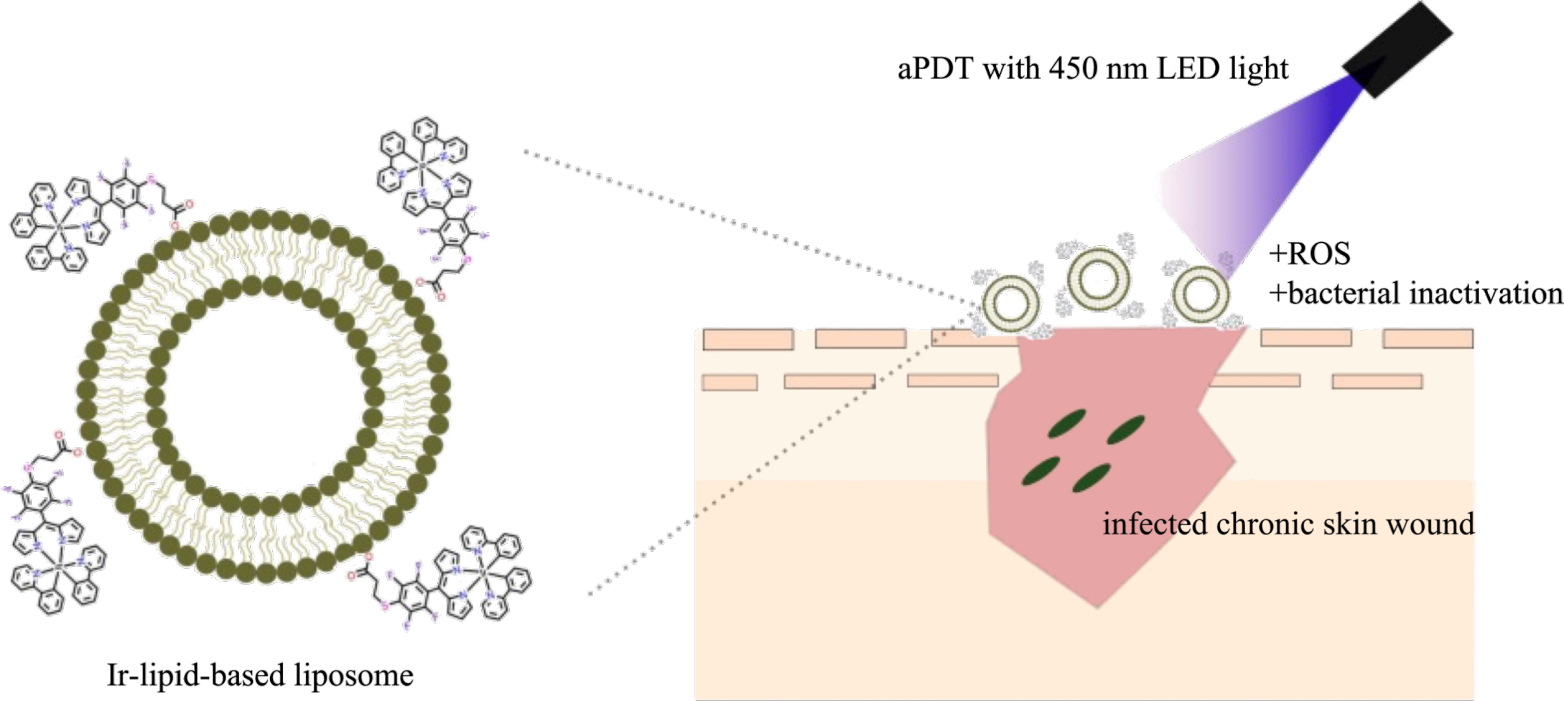
Schematic representation of the aPDT mechanism using Ir-lipid-based liposomes.

For example, the encapsulation of the Ir-based phospholipid conjugate **21** (Figure 5) in liposomes have been explored as an strategy to enhance antimicrobial performance while preserving host cell compatibility [141]. Liposomal formulations incorporating the Ir complex displayed superior photophysical properties compared to the free metallodrug, notably higher light absorption, and greater photoemission output, resulting in enhanced bactericidal activity against *S. aureus* (Figure 7). The Ir-complex liposomes interact efficiently with 450 nm LED light, leading to ROS generation and subsequent bacterial inactivation at the site of an infected chronic skin wound. Thus, liposomes emerged as the most promising candidates for the development safer and more effective aPDT agents.

#### Cobalt complexes

Numerous studies have highlighted the antibacterial capabilities of cobalt complexes, often noting their superior efficacy when cobalt ions are chelated to a ligand rather than used in their free ionic form [142]. Importantly, both the ligand structure and the redox potential of the complex are key determinants of stability and therapeutic potency [143].

Nano-based delivery systems have also been used to improve the stability and therapeutic efficacy of cobalt metalloantibiotics. For this purpose, the cobalt(III) complex **22** (Figure 5) was encapsulated into a biocompatible nanocarrier system based on poly(lactic-*co*-glycolic acid)-*block*-polyethylene glycol (PLGA-PEG) [144]. The resulting nanoconjugate exhibited efficient loading of the cobalt compound and demonstrated a pH-responsive release profile, with drug liberation increasing progressively under more acidic conditions. Specifically, cumulative release over 168 h reached ≈17% at physiological pH (7.4), ≈58% at pH 5.8, and ≈74% at pH 4.8, mimicking the acidic microenvironment of infected tissues. In vitro biological evaluations revealed that the nano-encapsulated Co(III) formulation showed superior antibacterial effects against *S. aureus* and *E. coli* compared to the free complex. These findings suggest that encapsulation into PLGA-PEG not only improves bioavailability but also enhances therapeutic action, opening opportunities for the application of cobalt-based agents in nanomedicine.

## Conclusion

Antimicrobial resistance continues to pose a critical threat to global health, undermining the effectiveness of conventional antibiotics and complicating the treatment of infectious diseases. In the search for more effective antibiotic drugs, metalloantibiotics have emerged as a novel class of antimicrobial agents with the potential to overcome existing resistance mechanisms. These compounds, with their unique modes of action and structural diversity, represent a valuable addition to the antimicrobial arsenal.

Nevertheless, the clinical translation of metalloantibiotics remains hindered by key challenges, including systemic toxicity, poor stability, and a lack of targeted delivery. In this context, recent advances in nanotechnology-based drug delivery systems, such as liposomes, polymeric nanoparticles, and mesoporous silica nanoparticles, offer promising strategies to mitigate these limitations (Table 1).

**Table 1:** Nanotechnology-based systems used for metalloantibiotic delivery.

Lipid-based nanoparticles		

	Characteristics	Examples

liposomes	- self-assembled lipid bilayer vesicles - encapsulate hydrophobic and hydrophilic drugs - highly biocompatible	[113,128,133,141]
solid lipid NPs	- solid lipid core matrix stabilized by emulsifiers - suitable for controlled release - improved stability compared to liposomes	[105–108]

Polymeric nanoparticles		

	Characteristics	Examples

polymersomes	- vesicles made from amphiphilic block copolymers - similar to liposomes but with greater structural stability and tunable properties	[124,127]
nanocapsules	- polymeric solid shell that surrounds a core-forming space - high encapsulation efficiency and biodegradability	[111,114,118,123,129,144]
polymeric micelles	- self-assembled structures formed by amphiphilic polymers - encapsulate hydrophobic drugs	[98,102,112]

Inorganic nanoparticles		

	Characteristics	Examples

silica nanoparticles (SiNPs)	- high surface area for drug loading - can be functionalized for targeting	[121]
quantum dots (QDs)	- nanosized semiconductor crystals - attach therapeutic molecules to their surface	[122]

These nanocarriers not only improve the stability and bioavailability of metalloantibiotics but also ameliorate their toxicity and enhance their antibacterial effects, resulting in much improved therapeutic indexes. In addition, some formulations enable site-specific drug release in response to infection-associated stimuli, while others reduce off-target effects by confining drug activity to the desired site of action.

Moving forward, research should emphasize the development of highly biocompatible and stimuli-responsive delivery platforms, as well as the investigation of synergistic effects through combination therapies. In-depth in vivo studies will be essential to validate preclinical findings and ensure translational potential. Moreover, the integration of computational modelling and machine learning may expedite the rational design of new metal-based complexes and optimize their delivery systems. These technologies can provide valuable insights into target interactions, predict pharmacokinetic behavior, and guide the development of safer, more effective therapeutic candidates.

Altogether, the convergence of metalloantibiotics and advanced drug delivery technologies opens new avenues for combating drug-resistant infections. In this regard, a multidisciplinary approach, bridging chemistry, nanotechnology, microbiology, and pharmacology will be pivotal in advancing this emerging field toward clinical application.

## Data Availability

Data sharing is not applicable as no new data was generated or analyzed in this study.
